# Professional practice environment and advanced nursing interventions associated with nurse-perceived quality of healthcare delivery in selected rural facilities in Saudi Arabia: a multisite cross-sectional correlational study

**DOI:** 10.3389/fpubh.2026.1950161

**Published:** 2026-10-29

**Authors:** Asrar S. Almutairi, Rayhanah R. Almutairi, Mohammed Almutairi, Hend N. Almutairi, Abdulaziz M. Alodhailah

**Affiliations:** 1 Department of Community and Psychiatric Mental Health Nursing, College of Nursing, Princess Nourah bint Abdulrahman University, Riyadh, Saudi Arabia; 2 Department of Medical-Surgical Nursing, College of Nursing, King Saud University, Riyadh, Saudi Arabia; 3 School of Archaeology and Anthropology, College of Arts and Social Sciences, The Australian National University, Canberra, ACT, Australia

**Keywords:** advanced practice nursing, health promotion, nursing practice environment, nursing workforce, perceived healthcare quality, rural nursing, Saudi Arabia

## Abstract

**Background:**

Rural Saudi health services face workforce shortages and geographical isolation, and nurses are often the principal providers of clinical care, patient education, and health promotion. This study examined whether the professional practice environment (RPPE) and engagement in advanced nursing interventions were associated with nurses' perceived quality of healthcare delivery (PQHD) in selected rural Saudi facilities.

**Methods:**

This cross-sectional correlational study is reported according to STROBE. A total of 187 nurses from three rural healthcare facilities in three Saudi regions (South, North, East; one purposively selected facility per region) were recruited using proportional stratified random sampling within region × role strata. An online survey included the validated Revised Professional Practice Environment (RPPE) scale and two study-specific scales (advanced nursing interventions; PQHD) assessed for content validity and internal consistency. Analyses used Pearson correlations, *t*-tests, one-way ANOVA, and multivariable linear regression (IBM SPSS v26).

**Results:**

PQHD was positively and significantly correlated with the RPPE total score (*r* = 0.56, *p* < 0.001) and with advanced nursing interventions (*r* = 0.47, *p* < 0.001); the two predictors were moderately intercorrelated (*r* = 0.55, *p* < 0.001). In the multivariable model, the RPPE total score (β = 0.45, *p* < 0.001) and advanced nursing interventions (β = 0.25, *p* < 0.001) remained independently associated with PQHD after adjustment for nursing role, years of experience, and site (region); the model explained 43% of the variance (*R*^2^ = 0.43, adjusted *R*^2^ = 0.40, *F*_(7, 179)_ = 19.07, *p* < 0.001). In bivariate analyses, nurse practitioners reported higher PQHD than registered nurses, male nurses reported slightly higher PQHD than female nurses, and PQHD did not differ by age group, education, years of experience, or region; neither difference survived Holm adjustment.

**Conclusion:**

A more favorable professional practice environment and more frequent engagement in advanced nursing interventions were independently associated with higher nurse-perceived quality of healthcare delivery in these rural Saudi facilities. Because the design is cross-sectional and the outcome is nurse-reported, causal and patient-level inferences cannot be drawn. These associations may inform efforts to strengthen rural practice environments and advanced practice roles, pending longitudinal, multi-source confirmation.

## 1 Introduction

The provision of quality healthcare in rural settings remains a global challenge, particularly where geographical, infrastructural, and workforce constraints intersect to limit access to essential services (1). In Saudi Arabia, characterized by vast distances, dispersed populations, and a centralized healthcare infrastructure, rural and remote communities frequently experience disparities in health outcomes relative to urban centers (2). These disparities are compounded by an uneven distribution of healthcare professionals, with rural areas often underserved in both the number and range of care providers. Within this context, nurses are frequently the frontline of healthcare delivery, assuming expansive, multidisciplinary, and often autonomous roles (3).

Globally, the role of nurses in rural health systems has attracted increasing attention because of their positioning at the intersection of clinical practice, public health, and community engagement (4). Unlike urban counterparts, rural nurses commonly function as generalists, delivering emergency response, chronic disease management, maternal and child health, patient education, and community health promotion, often with minimal specialist support (5, 6). This expanded scope requires advanced competencies in clinical decision-making, leadership, and cultural responsiveness (7). Yet rural nurses frequently work in environments marked by professional isolation, limited continuing education, and infrastructural deficiencies that impede optimal care delivery (8–10).

In Saudi Arabia, the Ministry of Health and allied stakeholders have prioritized expanding rural healthcare access under Vision 2030, including infrastructure development, mobile health units, and telemedicine (11). These initiatives, however, often underemphasise the strategic importance of nursing. Although Saudi nurses constitute a growing proportion of the national workforce, particularly in primary and community care, their contributions to rural healthcare delivery remain under-documented (12). Most existing literature addresses physician distribution or nursing workforce issues in tertiary hospitals (13).

Studies in Australia, Canada, and sub-Saharan Africa indicate that nurse-led models of care can improve accessibility, chronic disease outcomes, and patient satisfaction in rural and remote settings (14), and in hospital settings greater nurse autonomy has been associated with lower 30-day mortality and failure to rescue (15). Professional autonomy depends on organizational conditions such as shared leadership, inter- and intra-professional collaboration, and a healthy work environment (16). In Saudi Arabia these conditions are shaped by cultural and organizational factors, including gender norms and workforce composition (12), so the transferability of findings from other countries is uncertain.

In this study, advanced nursing interventions are clinical activities that go beyond routine, task-based care: complex assessment, protocol-driven clinical decisions, interdisciplinary care planning, independent patient education, escalation and referral, and medication titration where local policy permits. They are measured as how often a nurse performs them, regardless of job title. Advanced practice nursing, by contrast, refers to formally designated roles, such as the nurse practitioner, that require graduate preparation, credentialing, and an expanded, regulated scope of practice. A registered nurse may therefore perform advanced interventions without holding an advanced practice role, and the study examines these activities across all nursing roles.

The knowledge base lacks rigorous, data-driven evaluations of nurses' roles in rural Saudi healthcare (17). To our knowledge, no previous study in rural Saudi Arabia has examined whether the professional practice environment and advanced nursing interventions are independently associated with nurses' perceived quality of care after adjustment for role, experience, and region. This gap hinders evidence-based workforce planning and policy (18).

In rural settings, nurses are frequently the primary deliverers of patient education, health-literacy support, chronic-disease self-management coaching, and community health promotion; understanding the conditions under which they practice is therefore directly relevant to public health education and promotion. By examining nurses' perceptions of their practice environment, their engagement in advanced interventions, and their appraisal of care quality, this study aims to inform rural workforce development, continuing professional education, and health-promotion capacity-building.

In rural Saudi Arabia, primary care sites often operate with limited on-site diagnostics and intermittent specialist availability, increasing reliance on generalist nurses to coordinate acute care, chronic disease management, and patient education (19). Within this context, examining how the practice environment and advanced interventions relate to perceived care quality can identify practical targets for service improvement.

### 1.1 Objective

To examine whether nurses' perceptions of the professional practice environment (RPPE) and their engagement in advanced nursing interventions are associated with perceived quality of healthcare delivery (PQHD) among nurses in selected rural Saudi facilities, while adjusting for prespecified contextual covariates (nursing role, years of experience, and region).

### 1.2 Research questions

1. Are the professional practice environment and advanced nursing interventions independently associated with PQHD after adjustment for nursing role, years of experience, and region?
2. Does PQHD differ across demographic and working-condition variables (gender, age group, education, nursing role, years of experience, region)?

### 1.3 Rationale for emphasis variables

Nursing role, years of experience, and region were treated as key contextual factors because they relate to scope of practice and task mix (role), clinical expertise and decision latitude (experience), and service configuration/resource availability (region). They also defined the sampling strata (region × role) and were therefore prespecified as covariates and subgroup descriptors rather than primary predictors.

### 1.4 Hypothesis

A more favorable practice environment and greater engagement in advanced nursing interventions are associated with higher PQHD.

## 2 Materials and methods

### 2.1 Study design

This study employed a cross-sectional, descriptive correlational design to examine associations between the professional practice environment, advanced nursing interventions, and perceived quality of healthcare delivery (PQHD) in selected rural Saudi facilities. All variables were observed without manipulation; no causal inference was intended or claimed. Prespecified subgroup analyses compared PQHD by nursing role and years of experience, and role/experience/region were included as covariates in the multivariable model. The study is reported in accordance with the STROBE guideline for cross-sectional studies (Supplementary File S3).

### 2.2 Study setting and facility selection

Data were collected from rural healthcare facilities in three Saudi regions (South, North, East) (Supplementary Table S1). Facilities were purposively selected on prespecified criteria: (i) classification as a rural primary health center or rural general hospital by the Ministry of Health; (ii) a nursing establishment large enough to support stratified sampling; and (iii) administrative agreement to participate. Three facilities participated, one in each region. The South facility contributed 66 participants (54 registered nurses, six nurse practitioners, and six managers/supervisors), the North facility 65 (49, 4, and 12), and the East facility 56 (46, 4, and 6). Because each region was represented by a single facility, region-level and facility-level characteristics are inherently confounded; differences in PQHD between regions are therefore reported as site-level descriptive findings, and the region terms in the regression are interpreted as site indicators rather than as evidence of regional effects (see Section 5). Because facility selection was purposive rather than random, the sample represents the participating facilities rather than all rural nurses in Saudi Arabia, and regional inferences are correspondingly limited.

Participating sites comprised primary health centers and general hospitals providing first-contact care, stabilization, chronic disease follow-up, and referral coordination. Nursing teams ranged from diploma through baccalaureate to postgraduate preparation. Frontline registered nurses primarily delivered direct care, education, medication administration, and protocol-based escalation; postgraduate-prepared nurses, including nurse practitioners where deployed, undertook expanded assessment and led outreach/coordination; supervisory nurses focused on staffing, governance, and quality monitoring. Because each region contributed a single facility, the findings describe the three participating sites and should not be generalized to the regions in which they are located or to rural Saudi Arabia as a whole.

### 2.3 Sampling, power, and subgroups

Proportional stratified random sampling was used to reflect the distribution of nurses across geography and role while supporting planned subgroup comparisons. Strata were defined *a priori* as Region (South, North, East) × Role (registered nurse, nurse practitioner, nurse manager/supervisor), yielding nine strata, because exposure to autonomy, professional development, and advanced interventions plausibly varies by both region and role.

### 2.4 Sampling frame and selection

For each facility, Human Resources provided de-duplicated rosters listing licensure, role, and contact details. Within each stratum h, proportional allocation was applied:


$$\begin{array}{l} \text{n\_h }=\text{ n\_target }\times \;\left(\text{N\_h }÷\text{ Σ\_h N\_h}\right) \end{array}$$

where N_h is the stratum roster size, Σ_h N_h is the total eligible roster across strata, and n_target is the planned sample. Within strata, simple random sampling used a computer-generated random-number list. Prespecified replacement rules applied to non-responders (no response after two reminders over 14 days), intended to preserve proportional allocation. The participant flow and participation rate are reported in Section 2.7 (Target and final sample) and Figure 1. Supplementary Table S1 reports the completed sample for each of the nine strata.

**Figure 1:**
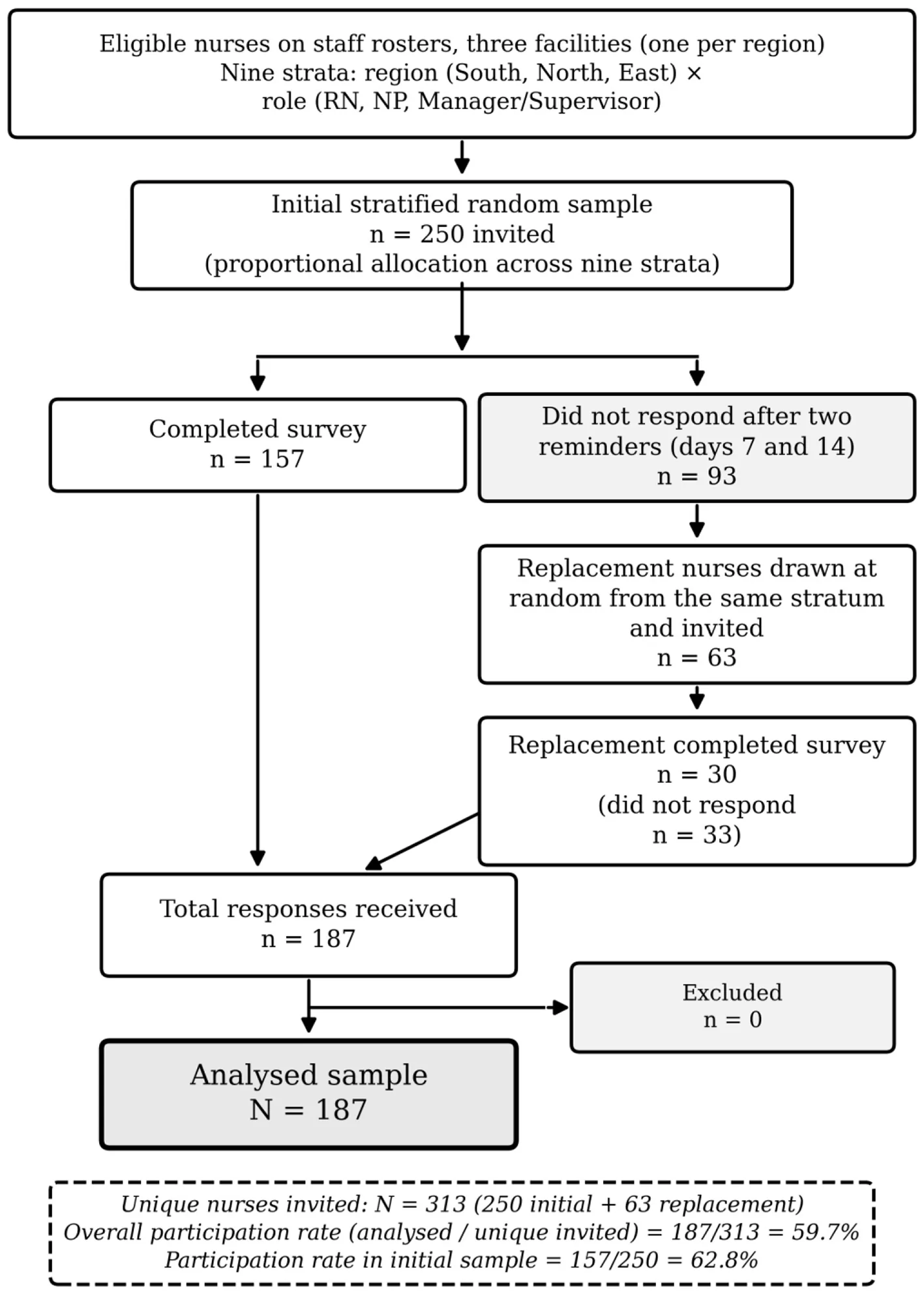
Participant flow through stratified sampling and survey completion. Nurses were sampled from nine region × role strata (South, North, East regions × registered nurse, nurse practitioner, nurse manager/supervisor roles) using proportional stratified random sampling. The initial random sample of 250 nurses yielded 157 completed surveys and 93 non-responders. 63 replacement nurses were selected at random from the corresponding strata and invited, of whom 30 completed the survey and 33 did not respond. No completed surveys were excluded. Of 313 unique nurses invited, 187 were analyzed (overall participation rate 59.7%; 62.8% within the initial sample). The analyzed sample by region × role stratum is reported in Supplementary Table S1.

### 2.5 Participants and eligibility

#### 2.5.1 Inclusion criteria

Licensed nurse in a participating rural facility; ≥1-year post-licensure experience; ability to complete the survey in Arabic or English. The ≥1-year criterion ensured participants had completed transition-to-practice and were familiar with local protocols and escalation pathways. Nurses in direct-care roles or in managerial/supervisory positions with clinical-governance responsibilities (e.g., staffing decisions, quality monitoring, escalation oversight) were eligible. Nursing role, including nurse practitioner status, was taken from the role designation on the Human Resources roster (see Section 2.4). In the participating sites, nurse practitioners were postgraduate-prepared nurses who undertook expanded assessment and led outreach and care coordination (Section 2.2).

#### 2.5.2 Exclusion criteria

Non-clinical administrative roles without clinical-governance responsibilities, and temporary agency staff without continuous placement.

### 2.6 A priori power

Power was computed for the primary analysis (a multiple linear regression of PQHD on the two main predictors [RPPE total, advanced nursing interventions] plus the prespecified covariates [nursing role, years of experience, region], comprising seven predictor terms), assuming two-tailed α = 0.05, power = 0.80, and a medium effect size *f*^2^ = 0.15. Under these assumptions the minimum required sample was *N* = 103 (G^*^Power 3.1).

### 2.7 Target and final sample

A target of *N* = 250 was set to maintain proportional representation across the nine strata, improve precision, and ensure adequate subgroup cell sizes. The initial stratified random sample comprised 250 nurses selected from eligible staff rosters across the nine region × role strata. Of these, 157 completed the survey and 93 did not respond after two reminders over 14 days. Under the prespecified within-stratum replacement procedure, 63 replacement nurses were then selected at random from the corresponding strata and invited, of whom 30 completed the survey and 33 did not respond. Replacement could not be completed for the remaining 30 initial non-responders because, in some strata, all eligible nurses on the roster had already been invited, and no further replacements were drawn after the data-collection window closed in November 2024. No completed surveys were excluded, so the 187 responses (157 initial and 30 replacement) formed the analytic sample. In total, 313 unique nurses were invited; the overall participation rate, defined as complete analysable responses divided by all unique nurses invited, was 59.7% (187/313), and the participation rate within the initial random sample was 62.8% (157/250). The participant flow is shown in Figure 1, and the analyzed sample by stratum in Supplementary Table S1. Although below the aspirational target, *N* = 187 exceeds the powered minimum (*N* = 103) and supports the primary association analyses. Power for subgroup comparisons was limited, particularly for the nurse-practitioner subgroup (*n* = 14), and subgroup results are interpreted with caution.

### 2.8 Prespecified subgroups

Role: registered nurse; nurse practitioner; nurse manager/supervisor. Years of experience: 1–4, 5–9, 10–14, and ≥15 years. These four bands were prespecified and used consistently in Tables 1, 2 and in the regression model. They are study-defined categories, chosen to provide adequate cell sizes for subgroup analysis; they are loosely informed by Benner's novice-to-expert framework (20), which describes stages of clinical skill acquisition but does not specify year-based cutoffs. Age group: 23–28, 29–36, 37–45, and ≥46 years. Age was recorded in these four categories on the questionnaire; the categories were study-defined to separate early-career, mid-career, and later-career nurses and were not derived from the sample distribution or from an external classification, which accounts for the unequal group sizes. In the regression, years of experience was entered as a single ordinal term (1 = 1–4, 2 = 5–9, 3 = 10–14, 4 = ≥15 years), so its coefficient represents the expected change in PQHD for a one-category increase in experience band, not for a fixed number of years. Because the bands are unequal in width and the top band is open-ended, this coding assumes equal spacing between adjacent categories; it was chosen *a priori* for parsimony given the small ≥15-year cell. A sensitivity model replacing the ordinal term with three dummy variables (reference = 1–4 years) left the main predictors essentially unchanged (RPPE β = 0.45; advanced nursing interventions β = 0.24), and no experience dummy was significant (Supplementary Table S5).

**Table 1:** Demographic characteristics of respondents (*N* = 187).

Variable	Category	*n*	%
Age (years)	23–28	27	14.4
	29–36	79	42.2
	37–45	57	30.5
	≥46	24	12.8
Gender	Male	68	36.4
	Female	119	63.6
Education	Diploma	28	15.0
	Bachelor's	100	53.5
	Master's	47	25.1
	Doctorate	12	6.4
Experience (years)	1–4	63	33.7
	5–9	57	30.5
	10–14	46	24.6
	≥15	21	11.2
Nursing role	Registered nurse	149	79.7
	Nurse practitioner	14	7.5
	Manager/supervisor	24	12.8

**Table 2:** Differences in PQHD by participant characteristics (bivariate) (*N* = 187).

Characteristic	Category (*n*, %)	PQHD mean (SD)	Test (*t*/*F*)	*p*	Effect size	*Post hoc* (adjusted)
Gender	Male (68, 36.4)	4.04 (0.37)	*t* = 2.33	0.021	*d* = 0.35	n/a
	Female (119, 63.6)	3.91 (0.35)				
Age	23–28 (27)	3.91 (0.37)	*F* = 0.37	0.774	η^2^ = 0.01	n/a
	29–36 (79)	3.97 (0.37)				
	37–45 (57)	3.99 (0.35)				
	≥46 (24)	3.93 (0.38)				
Education	Diploma (28)	3.94 (0.29)	*F* = 1.85	0.140	η^2^ = 0.03	n/a
	Bachelor's (100)	3.93 (0.39)				
	Master's (47)	4.06 (0.35)				
	Doctorate (12)	3.87 (0.25)				
Nursing role	Registered nurse (149)	3.93 (0.37)	*F* = 3.48	0.033	η^2^ = 0.04	NP > RN (*p* = 0.031); Mgr vs. RN, ns; NP vs. Mgr, ns
	Nurse practitioner (14)	4.19 (0.24)				
	Manager/supervisor (24)	4.01 (0.29)				
Experience	1–4 (63)	3.92 (0.39)	*F* = 0.50	0.684	η^2^ = 0.01	n/a
	5–9 (57)	4.00 (0.38)				
	10–14 (46)	3.97 (0.34)				
	≥15 (21)	3.94 (0.28)				
Region	South (66, 35.3)	4.01 (0.32)	*F* = 1.29	0.278	η^2^ = 0.01	n/a
	North (65, 34.8)	3.96 (0.41)				
	East (56, 29.9)	3.90 (0.35)				

Holm-adjusted p-values for the six comparisons: gender 0.12, nursing role 0.16, education 0.56, region 0.83, age group and years of experience 1.00. *Post hoc* comparisons (Tukey HSD) were run only for significant omnibus tests; Levene's test was non-significant for every characteristic. d, Cohen's d; η^2^, eta squared; NP, nurse practitioner; RN, registered nurse; Mgr, manager/supervisor; ns, not significant.

### 2.9 Nonresponse management

Two reminders (days 7 and 14) were issued; proportional within-stratum replacement limited differential nonresponse. Residual nonresponse bias is acknowledged in the Limitations.

### 2.10 Instruments

A structured, self-administered questionnaire comprised demographic items and three instruments. To distinguish validated from study-specific measures, the professional practice environment was assessed with the previously validated RPPE scale, whereas the Advanced Nursing Interventions and PQHD measures were developed or adapted for this study and evaluated only for content validity and internal consistency; they are not characterized as fully validated instruments.

#### 2.10.1 Translation and cultural adaptation

The English instruments were translated into Arabic and independently back-translated by two bilingual nurse experts; discrepancies were reconciled by a third bilingual expert to preserve conceptual and semantic equivalence. Content validity of the two study-specific instruments (Advanced Nursing Interventions and PQHD) was assessed by a panel of five experts in rural nursing, nursing education, and health-services research; the Scale-Content Validity Index averaged across items (S-CVI/Ave) was 0.91 across the two scales.

(1) Revised Professional Practice Environment (RPPE) scale. The 39-item Revised Professional Practice Environment scale (21) measures eight components of the professional practice environment: handling disagreement and conflict, leadership and autonomy in clinical practice, internal work motivation, control over practice, teamwork, communication about patients, cultural sensitivity, and staff relationships with physicians. Items are rated 1 (strongly disagree) to 4 (strongly agree). The primary environmental predictor was the RPPE total score (mean of items, range 1–4; maximum possible = 4.00), with higher scores indicating a more favorable environment. Internal consistency of the RPPE total score in this sample was high (Cronbach's α = 0.97). Component scores were not calculated or analyzed; only the total score was used.

(2) Advanced Nursing Interventions scale (study-specific, NREM-guided). Six items adapted from the “nursing process” component of the Nursing Role Effectiveness Model (22), with expert consultation, to reflect advanced rural nursing practices in the Saudi context. Items covered complex assessment, protocol-driven decisions, interdisciplinary planning, independent patient education, escalation/referral, and medication titration (where applicable under local policy). These activities were classed as advanced because they require independent clinical judgement and go beyond routine, task-based care (see Introduction). Participants rated how often they performed each activity on a 5-point frequency scale from 1 (rarely) to 5 (always); a composite mean (range 1–5) was computed. The medication titration item applied only where local policy permitted titration. Cronbach's α = 0.90; corrected item–total correlations 0.70–0.76. Full item wording appears in Supplementary Table S2. Only content validity and internal consistency were established; construct validity (convergent and discriminant) and test–retest reliability were not assessed.

(3) Perceived Quality of Healthcare Delivery (PQHD) scale (study-specific; dependent variable). Five items capturing nurses' perceptions of overall care quality, patient satisfaction, safety, continuity, and coordination, rated 1 (very poor)−5 (excellent); a composite mean (range 1–5) was computed. Cronbach's α = 0.73; corrected item–total correlations 0.46–0.53. Full item wording appears in Supplementary Table S2. PQHD reflects nurses' perceptions of care quality and is not a standardized, patient-reported, or objective clinical outcome. Although conceptually aligned with the Quality Nursing Care Scale and the Good Nursing Care Scale, it was not formally validated against them.

### 2.11 Data collection procedure

Data were collected July–November 2024 using a secure, web-based survey administered to nurses sampled via the stratified procedure. Randomly selected nurses received an individualized link by institutional email; two reminders (days 7 and 14) were issued, with proportional within-stratum replacements. Participants gave electronic informed consent on the survey's opening screen before any questions were shown. The questionnaire was available in Arabic and English. The platform enforced one response per link and recorded only study IDs; no direct identifiers were collected. Rules for handling partial responses are described in Section 2.6.

### 2.12 Data analysis

Data were analyzed using IBM SPSS Statistics v26. Continuous variables were summarized with means and SDs, categorical variables with frequencies and percentages. All 187 returned surveys were complete; no partial responses required listwise deletion, and *N* = 187 was analyzed for all models.

Regression assumptions were checked: linearity via partial-regression and residual-vs.-fitted plots; normality of residuals via Q–Q plots and the Shapiro–Wilk test (*W* = 0.99, *p* = 0.47); homoscedasticity via residual-vs.-fitted plots; multicollinearity via variance inflation factors (all VIF < 2, Table 3); and influential observations via Cook's distance and leverage. No influential observations were detected: all Cook's distance values were well below 1 (maximum = 0.04), and no case exceeded conventional leverage thresholds. Pearson correlations were used because bivariate linearity and approximate normality were supported; had these failed (non-normal residuals or clear non-linearity on inspection), Spearman correlations would have been used.

**Table 3:** Multiple linear regression predicting PQHD (*N* = 187).

Predictor	*B*	SE	β	*p*	95% CI for B	VIF
Main predictors						
RPPE (total)	0.43	0.07	0.45	< 0.001	[0.30, 0.56]	1.46
Advanced nursing interventions	0.16	0.04	0.25	< 0.001	[0.07, 0.25]	1.45
Nursing role (ref: RN)						
Nurse practitioner	0.26	0.08	0.19	0.001	[0.10, 0.41]	1.02
Manager/supervisor	0.15	0.06	0.14	0.020	[0.02, 0.27]	1.04
Years of experience (ordinal, per one-category increase)	0.03	0.02	0.07	0.206	[−0.01, 0.07]	1.03
Region/site (ref: east)						
North	0.09	0.05	0.12	0.091	[−0.01, 0.19]	1.45
South	0.14	0.05	0.19	0.006	[0.04, 0.24]	1.44

Model fit: F_(7, 179)_ = 19.07, p < 0.001; R^2^ = 0.43; adjusted R^2^ = 0.40.

The model contains seven predictor terms: RPPE total, advanced nursing interventions, two nursing-role dummies (reference = RN), years of experience (ordinal), and two region dummies (reference = east). Years of experience was coded 1 = 1–4, 2 = 5–9, 3 = 10–14, 4 = ≥15 years; its coefficient is the change in PQHD per one-category increase. Each region was represented by one facility, so the region terms are site indicators and cannot be interpreted as regional effects. β, standardized coefficient; CI, confidence interval; VIF, variance inflation factor.

Group differences in PQHD were tested with independent-samples *t*-tests or one-way Analysis of Variance (ANOVA). Homogeneity of variance was assessed with Levene's test; where violated, Welch's ANOVA was used. For every significant omnibus ANOVA, adjusted *post hoc* pairwise comparisons were conducted (Tukey HSD when variances were homogeneous, Games–Howell otherwise) and are reported in Table 2. The primary analysis was a single prespecified regression model; the six bivariate comparisons in Table 2 were secondary and exploratory, and Holm-adjusted *p*-values are reported alongside unadjusted values to account for multiple testing. Floor and ceiling effects were defined as more than 15% of respondents at the minimum or maximum possible score.

The primary inferential analysis was multiple linear (OLS) regression with PQHD as the dependent variable; the RPPE total score and advanced nursing interventions as the two prespecified main predictors; and nursing role (dummy-coded, reference = registered nurse), years of experience (ordinal), and region (dummy-coded, reference = East) as prespecified covariates. Covariates were selected on conceptual grounds (scope of practice, clinical expertise, service configuration), not on bivariate significance. Because each region was represented by one facility, the region dummies function as site fixed effects; the coefficients for the main predictors are therefore estimated within sites, and the region coefficients are interpreted as site-level differences. Nurses in the same facility may also share unmeasured influences, so responses may not be independent. Site fixed effects remove between-facility differences in mean PQHD but not any correlation among residuals within a facility. We therefore estimated the intraclass correlation (ICC) of PQHD by facility and, as sensitivity analyses, fitted a linear mixed model with a random intercept for facility and re-estimated the primary model with heteroscedasticity-consistent (HC3) standard errors (Supplementary Table S6). With only three facilities, the between-facility variance is estimated imprecisely and cluster-robust standard errors are not reliable, so these analyses test the robustness of the primary estimates rather than fully modeling clustering. A prespecified sensitivity analysis additionally adjusting for gender, age group, and education (Supplementary Table S4) and a second sensitivity analysis with years of experience dummy-coded (Supplementary Table S5) left the RPPE total score and advanced nursing interventions as the strongest independent correlates of PQHD, with negligible change in their coefficients. Statistical significance was set at two-tailed *p* < 0.05.

### 2.13 Ethical considerations

The study received ethical approval from the Institutional Review Board (IRB) at King Saud University (Approval No. 24-668, dated 13 April 2024). All participants gave electronic informed consent on the survey's opening screen before any questions were shown. Participation was voluntary and could be discontinued at any time; no incentives were offered. Email distribution lists were maintained separately from survey data; analyses used de-identified datasets labeled with study IDs only. All procedures adhered to the Declaration of Helsinki and national and institutional guidelines.

## 3 Results

### 3.1 Participant characteristics

Table 1 summarizes participant characteristics (*N* = 187). Participants were predominantly female and most commonly aged 29–36 years; most held a bachelor's degree. Registered nurses formed the large majority of the sample, and experience levels spanned early-career to senior practice. Full detail is presented in Table 1; percentages may not total 100 due to rounding.

### 3.2 Descriptive statistics

Perceptions of the practice environment, advanced interventions, and care quality were generally favorable (Table 4). There was no floor or ceiling effect on the outcome: no participant had the minimum PQHD score and 3 (1.6%) had the maximum, with scores spread across 3.20–5.00 (skewness 0.27). At item level, the proportion rating care as excellent ranged from 5.9 to 16.6%, so one item was slightly above the 15% threshold. Ceiling effects were also absent for the predictors (maximum score: RPPE 1.6%, advanced nursing interventions 6.4%).

**Table 4:** Descriptive statistics for study variables (*N* = 187).

Variable (scale range)	Mean	SD	Minimum	Maximum
RPPE—total score (1–4)	3.24	0.38	2.26	4.00
Advanced nursing interventions (1–5)	3.99	0.56	2.17	5.00
PQHD—total score (1–5)	3.96	0.36	3.20	5.00

Scale ranges are shown in parentheses. RPPE, Revised Professional Practice Environment; PQHD, perceived quality of healthcare delivery. Scores are item means.

### 3.3 Correlations

Both the RPPE total score and advanced nursing interventions were significantly and positively correlated with PQHD (RPPE: *r* = 0.56; advanced interventions: *r* = 0.47; both *p* < 0.001), and the two predictors were moderately intercorrelated (*r* = 0.55, *p* < 0.001) (Table 5).

**Table 5:** Pearson correlation matrix (*N* = 187).

Variable	1	2	3
1. RPPE (total)	n/a	0.55^***^	0.56^***^
2. Advanced Nursing Interventions	0.55^***^	n/a	0.47^***^
3. PQHD (total)	0.56^***^	0.47^***^	n/a

^***^p < 0.001 (two-tailed).

### 3.4 Bivariate differences in PQHD

PQHD differed by gender and nursing role but not by age group, education, years of experience, or region (Table 2). Male nurses reported slightly higher PQHD than female nurses (4.04 vs. 3.91; *t*_(185)_ = 2.33, *p* = 0.021, Cohen's *d* = 0.35). PQHD differed by nursing role (*F*_(2, 184)_ = 3.48, *p* = 0.033, η^2^ = 0.04). Because Levene's test was non-significant (*p* = 0.12), Tukey HSD comparisons were used: nurse practitioners (mean PQHD = 4.19) scored higher than registered nurses (3.93; *p* = 0.031), whereas managers/supervisors (4.01) did not differ from registered nurses (*p* = 0.59) or from nurse practitioners (*p* = 0.30). The nurse practitioner–registered nurse difference was 0.25 points (Tukey 95% CI 0.02–0.49), estimated from 14 nurse practitioners. Differences by age group (*F*_(3, 183)_ = 0.37, *p* = 0.77), education (*F*_(3, 183)_ = 1.85, *p* = 0.14), years of experience (*F*_(3, 183)_ = 0.50, *p* = 0.68), and region (*F*_(2, 184)_ = 1.29, *p* = 0.28) were not significant. After Holm adjustment for the six comparisons, neither the gender difference (adjusted *p* = 0.12) nor the role difference (adjusted *p* = 0.16) remained significant. These descriptive differences are reported without causal interpretation.

### 3.5 Multivariable regression

In the multivariable OLS model (Table 3), the RPPE total score and advanced nursing interventions were each independently associated with PQHD after adjustment for nursing role, years of experience, and region. The RPPE total score showed the larger standardized association (β = 0.45), followed by advanced nursing interventions (β = 0.25). Nurse practitioners (*B* = 0.26, *p* = 0.001) and managers/supervisors (*B* = 0.15, *p* = 0.020) reported higher PQHD than registered nurses, whereas years of experience was not associated with PQHD (*B* = 0.03 per one-category increase, *p* = 0.21). PQHD was higher at the South site than at the East site after adjustment (*B* = 0.14, *p* = 0.006), although the unadjusted regional difference was not significant; because each region was represented by a single facility, this term reflects a site-level difference and cannot be attributed to region. The covariates alone explained 5% of the variance in PQHD, and adding the two main predictors increased *R*^2^ by 0.37. VIF values ranged from 1.02 to 1.46, and no influential observations were detected (maximum Cook's distance = 0.04). Clustering by facility was negligible: the ICC for PQHD was 0.005 (0.044 for residuals of the model without site terms). In the random-intercept mixed model, estimates were nearly identical to the primary model (RPPE *B* = 0.43, advanced nursing interventions *B* = 0.16, nurse practitioner *B* = 0.26; all *p* ≤ 0.001), and HC3 standard errors did not change any conclusion (Supplementary Table S6).

## 4 Discussion

This cross-sectional study found that a more favorable professional practice environment and more frequent advanced nursing interventions were each independently associated with higher nurse-perceived quality of healthcare delivery in the participating rural facilities, associations that persisted after adjustment for role, experience, and region. RPPE component scores were not analyzed separately, so the discussion centers on the two prespecified predictors. The associations were of moderate magnitude and stable across bivariate and multivariable analyses.

The association between the practice environment and PQHD is consistent with international nursing research showing that supportive work environments are foundational to perceived care quality and safety. Environments characterized by autonomy, effective teamwork, and access to professional development have been associated with higher nurse-reported quality, reduced missed care, and improved patient experience (23, 24). In rural contexts, where nurses often operate with fewer on-site resources and reduced immediate physician availability, the practice environment may be especially influential (25); our findings extend this literature to rural Saudi Arabia. Higher autonomy and reliable escalation architecture have repeatedly been tied to timely decision-making and perceived safety at the point of care (24, 26).

Advanced nursing interventions showed a smaller, but still independent, association with PQHD than the practice environment, which is consistent with the Nursing Role Effectiveness Model, wherein nursing processes (assessment, education, coordination, escalation) link organizational structures to care outcomes (27, 28). In rural systems, advanced interventions often compensate for limited specialist access and delayed referrals, positioning nurses as central decision-makers in the care trajectory. The observed association is compatible with, but does not establish, the potential importance of enabling nurses to practice within their full scope (25). Because both predictors and the outcome were reported by the same nurses at a single time point, part of this association may reflect common-method variance rather than a relationship with objectively measured quality (see Section 5). The proportion of PQHD variance explained by the full model (43%) should therefore be interpreted cautiously: shared-respondent and single-instrument measurement can inflate associations among self-reported constructs, and how these associations relate to objectively measured quality cannot be determined from these data.

Several contextual factors may attenuate associations relative to urban or tertiary settings. Resource variability across rural regions (limited diagnostics, intermittent specialist availability, transport constraints) can compress PQHD variance (25). The inclusion of managers/supervisors introduces heterogeneity in vantage point, as managerial appraisals may emphasize governance rather than bedside phenomena (29). Unit-level evidence links a higher proportion of experienced nurses to fewer medication errors and falls (30), but PQHD did not differ across experience bands, either before or after adjustment. Education was not associated with PQHD, consistent with a study of Malaysian hospital nurses that found no association between nurses' educational level and quality of care (31). Nurse practitioners reported higher PQHD than registered nurses, and the difference persisted after adjustment. This finding should be treated as exploratory: it rests on 14 nurse practitioners, its confidence interval approaches zero (0.02–0.49), and the bivariate role difference was not significant after adjustment for multiple comparisons. The analysis does not show that role matters more than experience or education; these were measured differently and their effects were not compared directly. The small bivariate gender difference (male > female) was not retained when gender was added to the adjusted model (Supplementary Table S4). The higher adjusted PQHD at the South site should be read as a site-level finding; with one facility per region, it cannot be separated from facility characteristics such as staffing, leadership, or service mix.

### 4.1 Implications for public health education and promotion

From a public health education and promotion perspective, rural nurses are frequently the primary deliverers of patient education, health-literacy support, and community health promotion. The observed associations suggest that strengthening supportive practice environments may enhance nurses' capacity to perform these health-promotion functions, an outcome that these cross-sectional, nurse-reported data can only hypothesize and that requires confirmation with patient-level and objective indicators.

Practically, the observed association between advanced nursing interventions and nurse-perceived quality supports exploring, where locally appropriate and evidence-based, expanded advanced practice roles in underserved rural areas. Professional development was not measured or tested as a separate factor in this study. Equitable access to in-service training, simulation, and tele-education is therefore a recommendation based on the wider literature and the geographic constraints of rural practice, not a finding of this study, and it requires evaluation. Aligned with Vision 2030, incorporating rural nursing development into health-system strengthening is a plausible, testable direction rather than a demonstrated effect.

## 5 Strengths and limitations

This study has several strengths: it addresses an understudied rural context, includes participants from multiple facilities, uses probability-based sampling within strata, and reports effect sizes and confidence intervals.

Limitations should guide interpretation. First, the design is cross-sectional and observational, precluding causal inference; reverse or bidirectional relationships cannot be excluded. Second, because predictor and outcome data were collected from the same respondents via a single self-report survey at one time point, common-method variance may have inflated the observed correlations and model *R*^2^; the relationship with objectively measured quality is unknown. Third, the outcome (PQHD) is a nurse-reported perception, not a patient-reported or clinical measure, and is susceptible to social-desirability bias; triangulation with patient experience and facility-level indicators was not possible. Fourth, two of the three instruments (Advanced Nursing Interventions and PQHD) were developed or adapted for this study and evaluated only for content validity and internal consistency; their construct validity is unestablished, the internal consistency of the PQHD scale in this sample was acceptable but modest (α = 0.73), and related findings are preliminary. Fifth, facilities were purposively selected and only three of five macro-regions were sampled, limiting generalizability; because each region was represented by a single facility, region and facility effects are confounded, so regional differences may reflect facility-specific characteristics, are reported descriptively only, and should not be generalized to the regions in which the facilities are located. Sixth, the inclusion of managers/supervisors alongside frontline nurses introduces heterogeneity in vantage point that may attenuate contrasts even after adjustment. Seventh, voluntary web-based recruitment carries potential selection and nonresponse bias (overall participation rate 59.7%; replacement could not be completed for 30 initial non-responders, so stratum proportions in the analytic sample may depart from the planned allocation), and subgroup power, especially for nurse practitioners (*n* = 14), was limited. Finally, with only three facilities, facility-level clustering could not be modeled reliably; site indicators were included as fixed effects, and although the estimated clustering was negligible and a random-intercept model gave the same results, residual within-facility correlation cannot be excluded, and nurses with < 1 year of experience were excluded by design, limiting applicability to very early-career staff.

## 6 Conclusion

In this multisite cross-sectional study of nurses in selected rural Saudi facilities, a more favorable professional practice environment and more frequent advanced nursing interventions were independently associated with higher nurse-perceived quality of healthcare delivery, after adjustment for role, experience, and region. Nurse practitioners reported higher perceived quality than registered nurses, but the finding is exploratory: it rests on 14 nurse practitioners, and the bivariate role difference did not survive correction for multiple comparisons; years of experience was not associated with perceived quality. Because the design is cross-sectional and the outcome is a nurse-reported perception rather than an objective patient or clinical outcome, these findings identify associations rather than causal effects.

These associations are consistent with, but do not by themselves justify, investment in supportive rural practice environments and, where locally appropriate and evidence-based, expanded advanced practice roles. Continuing professional education was not examined in this study and requires separate evaluation. For public health education and promotion, strengthening the conditions under which rural nurses deliver patient education and health promotion is a plausible lever for improving care that warrants direct evaluation. Future research should use longitudinal or mixed-methods designs, larger and more representative samples, fully validated instruments, and objective, patient-reported quality indicators to test whether these associations reflect causal relationships.

## Data Availability

The raw data supporting the conclusions of this article will be made available by the authors, without undue reservation.
