# Supplementary material for: Professional practice environment and advanced nursing interventions associated with nurse-perceived quality of healthcare delivery in selected rural facilities in Saudi Arabia: a multisite cross-sectional correlational study
Source: Front Public Health. 2026 Oct 29;14:1950161. doi: 10.3389/fpubh.2026.1950161 (PMC13754894; doi:10.3389/fpubh.2026.1950161)
Supplement: Supplementary file 1 [file Table_1.doc]

**Supplementary File S3. STROBE checklist for cross-sectional studies**

*Professional Practice Environment and Advanced Nursing Interventions Associated with Nurse-Perceived Quality of Healthcare Delivery in Selected Rural Facilities in Saudi Arabia: A Multisite Cross-Sectional Correlational Study*

| **Section/Topic** | **Item** | **Recommendation (cross-sectional studies)** | **Where reported in the manuscript** |
| --- | --- | --- | --- |
| **Title and abstract** | | | |
| Title and abstract | 1a | Indicate the study design with a commonly used term in the title or the abstract | Title ("Multisite Cross-Sectional Correlational Study"); Abstract, Methods |
|  | 1b | Provide an informative and balanced summary of what was done and what was found | Abstract |
| **Introduction** | | | |
| Background/rationale | 2 | Explain the scientific background and rationale for the investigation | Section 1, Introduction |
| Objectives | 3 | State specific objectives, including any prespecified hypotheses | Section 1: Objective, Research questions, Hypothesis |
| **Methods** | | | |
| Study design | 4 | Present key elements of study design early in the paper | Section 2.1 |
| Setting | 5 | Describe the setting, locations, and relevant dates, including periods of recruitment, exposure, follow-up, and data collection | Sections 2.2 (facilities, one per region) and 2.5 (data collection July–November 2024) |
| Participants | 6a | Give the eligibility criteria, and the sources and methods of selection of participants | Section 2.2 (facility selection and description); Section 2.3: Sampling frame and selection; Participants and eligibility (including identification of nurse practitioners) |
| Variables | 7 | Clearly define all outcomes, exposures, predictors, potential confounders, and effect modifiers; give diagnostic criteria, if applicable | Section 2.4 (PQHD outcome; RPPE and Advanced Nursing Interventions predictors); Section 2.3 (subgroup definitions); Section 2.6 (prespecified covariates) |
| Data sources/measurement | 8 | For each variable of interest, give sources of data and details of methods of assessment; describe comparability of assessment methods if there is more than one group | Section 2.4 (RPPE components and scoring; response format of study-specific scales; translation and back-translation; content validity; reliability); Section 2.5; Supplementary Table S2 |
| Bias | 9 | Describe any efforts to address potential sources of bias | Section 2.3 (stratified random sampling, replacement, nonresponse management); Section 2.4 (validated RPPE scale); Section 5 (common-method, social-desirability, nonresponse, and site confounding) |
| Study size | 10 | Explain how the study size was arrived at | Section 2.3: A priori power; Target and final sample |
| Quantitative variables | 11 | Explain how quantitative variables were handled in the analyses; if applicable, describe which groupings were chosen and why | Section 2.3 (study-defined age and experience categories; ordinal coding of experience); Section 2.4 (item-mean scale scores) |
| Statistical methods | 12a | Describe all statistical methods, including those used to control for confounding | Section 2.6 |
|  | 12b | Describe any methods used to examine subgroups and interactions | Section 2.6 (t-tests, one-way ANOVA, Tukey HSD/Games–Howell rule, Holm adjustment for the six exploratory comparisons); Section 3.4; Table 4 and note |
|  | 12c | Explain how missing data were addressed | Section 2.6 (all 187 surveys complete; no missing data) |
|  | 12d | If applicable, describe analytical methods taking account of sampling strategy | Sections 2.3 and 2.6 (proportional allocation; site indicators as fixed effects; intraclass correlation, random-intercept mixed model, and HC3 standard errors for facility clustering); Supplementary Table S6 |
|  | 12e | Describe any sensitivity analyses | Sections 2.3 and 2.6; Supplementary Tables S4, S5, and S6 |
| **Results** | | | |
| Participants | 13a | Report numbers of individuals at each stage of study | Section 2.3: Target and final sample; Figure 1; Supplementary Table S1 (overall flow and completed sample by stratum) |
|  | 13b | Give reasons for non-participation at each stage | Section 2.3 (non-response after two reminders; reason replacement was incomplete; no exclusions); Figure 1 |
|  | 13c | Consider use of a flow diagram | Figure 1 |
| Descriptive data | 14a | Give characteristics of study participants (demographic, clinical, social) and information on exposures and potential confounders | Section 3.1, Table 1; Section 3.2, Table 2; Supplementary Table S1 |
|  | 14b | Indicate number of participants with missing data for each variable of interest | Section 2.6 (none) |
| Outcome data | 15 | Report numbers of outcome events or summary measures | Section 3.2, Table 2 (including floor and ceiling check) |
| Main results | 16a | Give unadjusted estimates and, if applicable, confounder-adjusted estimates and their precision (e.g., 95% confidence interval); make clear which confounders were adjusted for and why | Section 3.3, Table 3 (unadjusted); Section 3.5, Table 5 (adjusted, 95% CI); Section 2.6 (covariate rationale) |
|  | 16b | Report category boundaries when continuous variables were categorized | Section 2.3; Tables 1 and 4 |
|  | 16c | If relevant, consider translating estimates of relative risk into absolute risk for a meaningful time period | Not applicable (continuous outcome) |
| Other analyses | 17 | Report other analyses done (e.g., analyses of subgroups and interactions, and sensitivity analyses) | Section 3.4, Table 4; Section 3.5; Supplementary Tables S4, S5, and S6 |
| **Discussion** | | | |
| Key results | 18 | Summarise key results with reference to study objectives | Section 4, first paragraph |
| Limitations | 19 | Discuss limitations of the study, taking into account sources of potential bias or imprecision; discuss both direction and magnitude of any potential bias | Section 5 |
| Interpretation | 20 | Give a cautious overall interpretation of results considering objectives, limitations, multiplicity of analyses, results from similar studies, and other relevant evidence | Sections 4 and 4.1; Section 6 |
| Generalisability | 21 | Discuss the generalisability (external validity) of the study results | Section 2.2; Section 5 (fifth limitation) |
| **Other information** | | | |
| Funding | 22 | Give the source of funding and the role of the funders for the present study and, if applicable, for the original study on which the present article is based | Funding statement (Ongoing Research Funding Program, ORFFT-2026-1146, King Saud University; role of the funder stated) |

*Locations refer to section, table, and figure numbers in the revised manuscript (R1). Checklist items are based on the STROBE Statement (von Elm E, et al. Lancet 2007;370:1453–1457; www.strobe-statement.org), available under a Creative Commons Attribution licence.*
